# Supplementary material for: CoCo: RNA-seq read assignment correction for nested genes and multimapped reads
Source: Bioinformatics. 2019 May 29;35(23):5039–47. doi: 10.1093/bioinformatics/btz433 (PMC6901076; doi:10.1093/bioinformatics/btz433)
Supplement: btz433_Supplementary_Data [file btz433_supplementary_data.zip › btz433-Suppl_data/Supplementary_Data.pdf]

Supplemental Material

CoCo: RNA-seq Read Assignment Correction for Nested Genes and Multimapped Reads

Gabrielle Deschamps-Francoeur, Vincent Boivin, Sherif Abou Elela and Michelle S Scott

Supplementary Table 1: Proportion of nested genes of different biotype in human

Biotype	Total genes	Nested genes	Proportion of nested genes
**miRNA**	1567	938	0.60
**scaRNA**	53	30	0.57
**snRNA**	1899	642	0.34
**snoRNA**	962	592	0.62
**tRNA**	628	141	0.23
**lincRNA**	7532	253	0.034

Supplementary Table 2: Proportion of host genes of different biotype in human

Biotype	Total genes	Host genes to small RNAs	Proportion of host genes
**Protein coding**	20286	1430	0.070
**lncRNA**	14668	305	0.021
**Pseudogene**	14568	103	0.0071

Supplementary Table 3: Parameter values used for read assignment tools considered

Tool	Pipeline name in text	Shared parameter values	TGIRT-seq additional parameters [b,c]	Viral RT Ribodepleted additional parameters [c]	Reference [d]
CoCo	CoCo	-t 24<br>(-C, --largestOverlap, -B, --minOverlap 10, [-M]) [a]	-s 1 -p	-p	This paper
featureCounts	featureCounts_standard	--minOverlap 10 --largestOverlap -C -T 24	-s 1 -p	-p	Liao et al 2013
featureCounts	featureCounts_optimized	--minOverlap 10 --largestOverlap -C -T 24 -O -M --fraction	-s 1 -p	-p	Liao et al 2013
RSEM	RSEM	convert-sam-for-rsem -p 24 &&<br>rsem-calculate-expression -p 24 --alignments	--strand-specific --paired-end	--paired-end	Li et al 2010
HTSeq	HTSeq-count	-f sam -r name -m intersection-strict	--stranded=yes		Anders et al 2015
Cufflinks	Cufflinks	-p 24 --GTF			Trapnell et al 2012
STAR	STAR	--runMode alignReads<br>--runThreadN 46<br>--quantMode GeneCounts<br>--readFilesCommand zcat<br>--outReadsUnmapped Fastx<br>--outFilterType BySJout<br>--outStd Log<br>--outSAMunmapped None<br>--outSAMtype BAM SortedByCoordinate<br>--outSAMprimaryFlag AllBestScore<br>--alignIntronMax 1250000			Dobin and Gingeras 2016

[a] CoCo internally uses featureCounts with the parameters in parentheses. The parameter -M is only specified when assigning multimapped reads.
[b] -s 1 is used to specify that the library is stranded.
[c] -p is for paired-end reads.

[d] References

Anders, S., Pyl, P.T. and Huber, W. (2015) HTSeq--a Python framework to work with high-throughput sequencing data, *Bioinformatics*, **31**, 166-169.

Dobin, A. and Gingeras, T.R. (2016) Optimizing RNA-Seq Mapping with STAR, *Methods Mol Biol*, **1415**, 245-262.

Li, B., *et al.* (2010) RNA-Seq gene expression estimation with read mapping uncertainty, *Bioinformatics*, **26**, 493-500.

Liao, Y., Smyth, G.K. and Shi, W. (2013) The Subread aligner: fast, accurate and scalable read mapping by seed-and-vote, *Nucleic Acids Res*, **41**, e108.

Trapnell, C., *et al.* (2012) Differential gene and transcript expression analysis of RNA-seq experiments with TopHat and Cufflinks, *Nat Protoc*, **7**, 562-578.

Supplementary Table 4: Primers used for droplet digital PCR validation. The primer pairs used in Figure 3 are listed for each gene tested.

Gene name	Forward Sequence	Reverse Sequence
RN7SK	CGGTCTTCGGTCAAGGGTATACG	AGCGCCTCATTTGGATGTGTCT
RN7SL2	AGGTCGGAAACGGAGCAGGTC	CGGGGTCTCGCTATGTTGCT
SNORA44	GGGCTGTGGCTGGTCATAGC	AAAGCTGAGTGGCAGCTTGCAG
SNORA63	TAAGTGCTGTGTTGTCGTTCCCC	TATGAGACCAAGCGTCCCTGGC
SNORA64	AGTTGCACTTGGCTTCACCCG	GCACCCCTCAAGGAAAGAGAGG
SNORA68	GAATCACTGTTTCTTATAGCGGTGGTT	AAATTCACTTTGAGGGGCACGG
SNORD16	AATTTGCGTCTTACTCTGTTCTCAGC	TCAGTAAGAATTTTCGTCAACCTTCTGTAC
SNORD32A	AACATTCACCATCTTTCGTTTGAGTCTCAC	GTCTCAGAGCGGTGCATGGG
SNORD88C	AGCACTGGGCTCTGATCACCC	CCTCAGACCCCCAGGTGTCAA

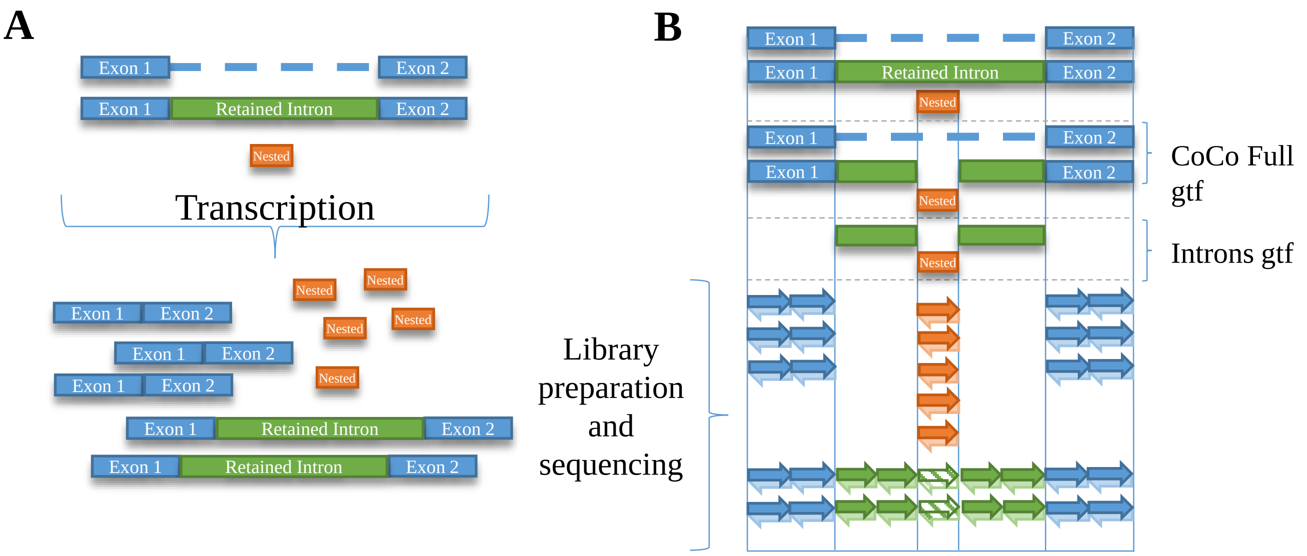

C

Feature	Feature length	CoCo Full gtf	CoCo introns	Number of reassigned reads	Final counts
Nested gene	x	7 = 5 + 2	7 = 5 + 2	- 8 * x/4x = -2	7 - 2 = 5
Overlapping intron (non-overlapping portion)	4x	NA	8	8 * x/4x = 2	NA
Host gene	y	28 = 20 + 8	NA	NA	28 + 2 = 30

Supplementary Figure 1 : Description of CoCo background correction for nested genes. (A) Genomic loci encoding multiple genes (ie nested genes) can produce transcripts from different genes containing the same sequence. In the example, the sequence of transcripts from the nested gene (orange) is identical to part of the sequence of a retained intron (green) of a host gene transcript. (B) Sequencing reads for this group of transcripts can thus come from the exons (blue arrows), retained introns (green arrows) or nested gene transcripts (orange arrows). However, a fraction of the reads in the region in which the nested gene overlaps the retained intron likely originate from the retained intron and not the nested gene (stripped green arrows). (C) To attribute the reads between the host gene and nested gene transcripts appropriately, the context of the nested gene is considered and used as a background to be subtracted from the total read counts attributed to the nested gene. In the example presented, the nested gene is encoded in a retained intron. The portion of the retained intron not overlapping the nested gene is covered by two reads over it length, and thus it is assumed that the same proportion of reads relative to its length should be assigned to the retained intron in the region overlapping the nested gene. Thus reads are reassigned from the nested gene to the retained intron.

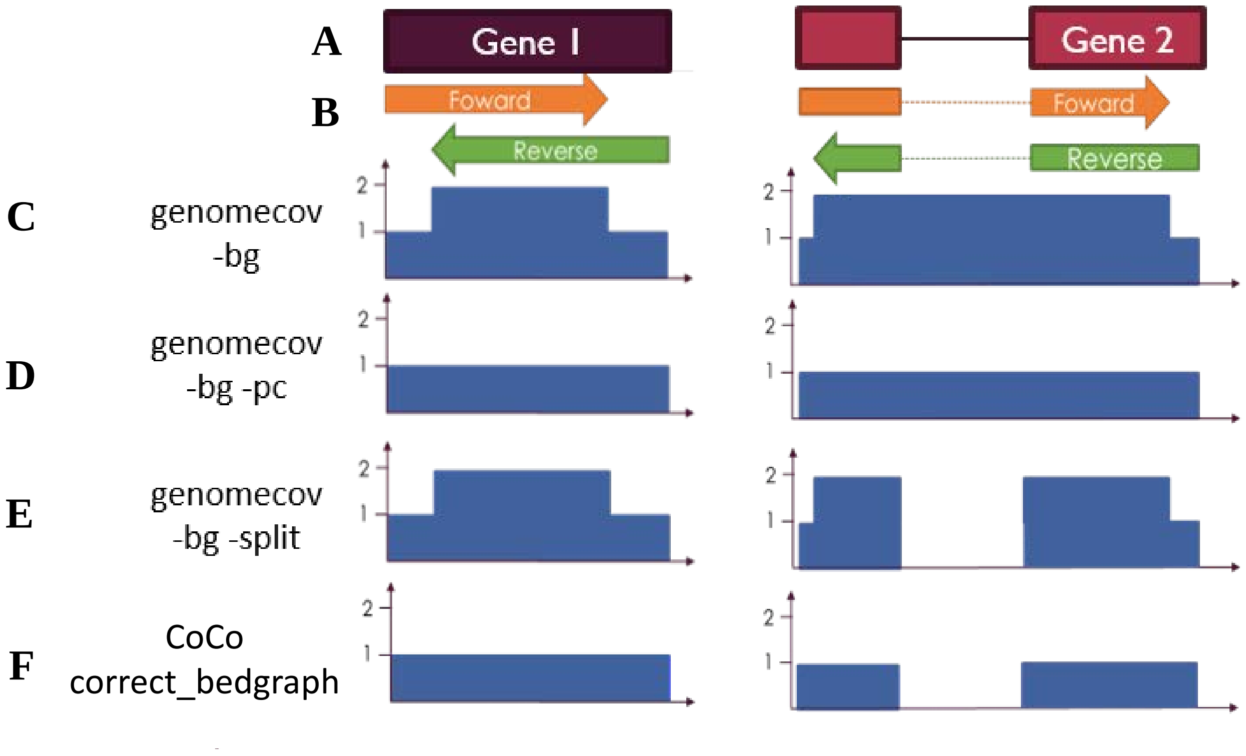

Supplementary Figure 2 : Result of CoCo's correct_bedgraph module. (A) Representation of two genes, consisting of one (Gene1) or two (Gene 2) exons. (B) Representation of a read pair aligned to each gene. The read pair aligned to Gene 2 includes a splice junction. (C-F) Graphical representations of the aligned read pairs using (C-E) Bedtools genomecov with specified options and (F) CoCo's correct_bedgraph module. When a read pair doesn't contain a splice junction, the abundance is doubled where the members of the pair overlap in C and E, while the members of the pair are counted as one in D and F. When the read pair contains a splice junction, the members of the pair are individually counted in C and E. C and D report coverage in the intron, while E and F don't. Finally, in F, the pair counts as only one and the intron isn't covered.

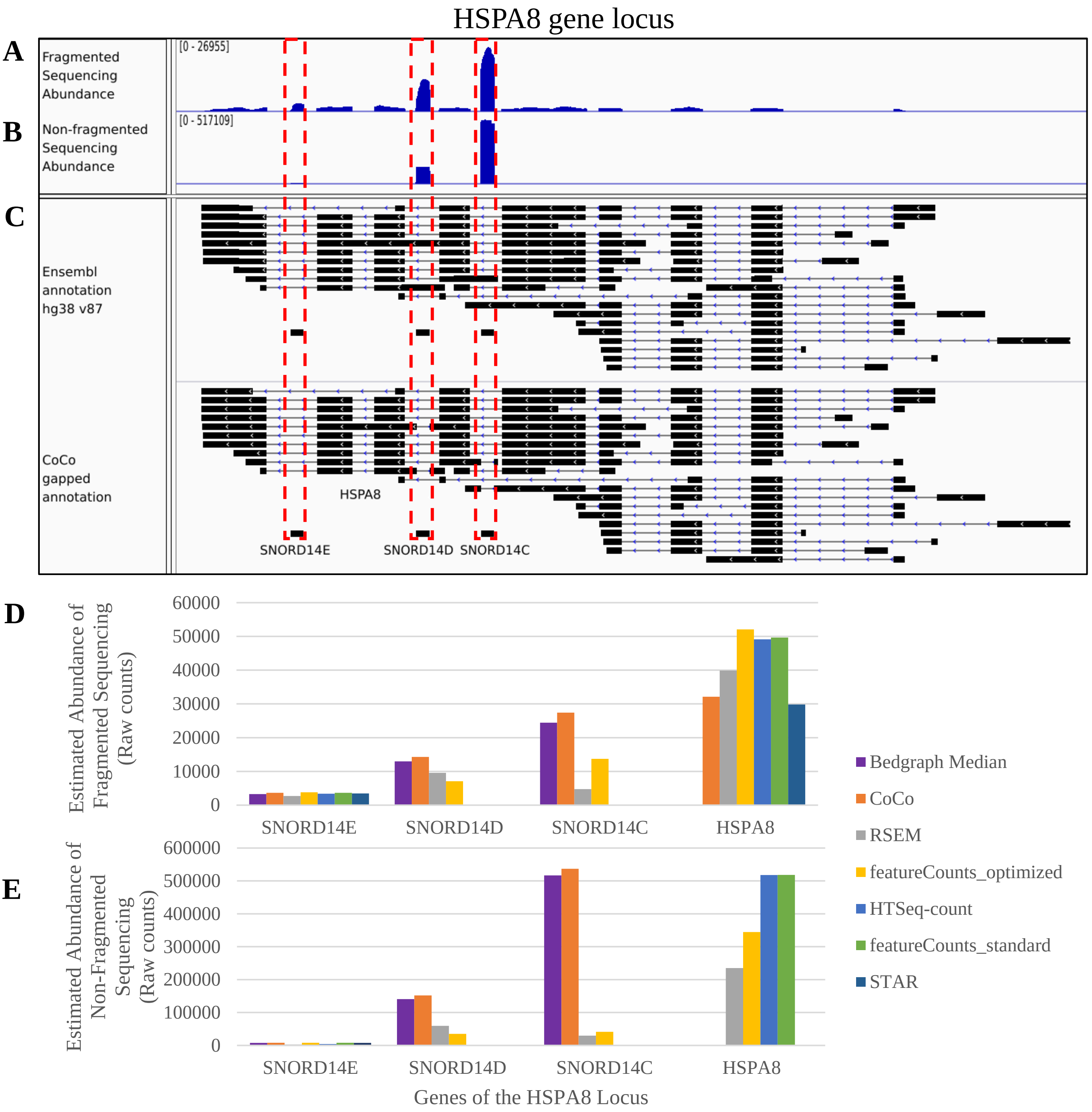

Supplementary Figure 3: **Example of nested C/D snoRNAs in TGIRT-seq fragmented and non-fragmented datasets.** (A,B) Bedgraph screenshot of the HSPA8 gene locus which encodes the coding gene HSPA8 and the nested box C/D snoRNAs SNORD14E, SNORD14D and SNORD14C. The nested genes are highlighted in dashed red boxes. The upper track (panel A) is from a fragmented sequencing library while the lower track (panel B) is from a non-fragmented sequencing library. Panel C shows the annotation tracks including the original Ensembl annotation and the CoCo gapped annotation highlighting nested genes. (D, E) Raw counts obtained using the different read assignment tools considered compared to the bedgraph median values calculated, for the fragmented (D) and the non-fragmented (E) datasets. The bedgraph median is only given for the small non-coding RNAs since it is not representative of the raw counts for longer genes.

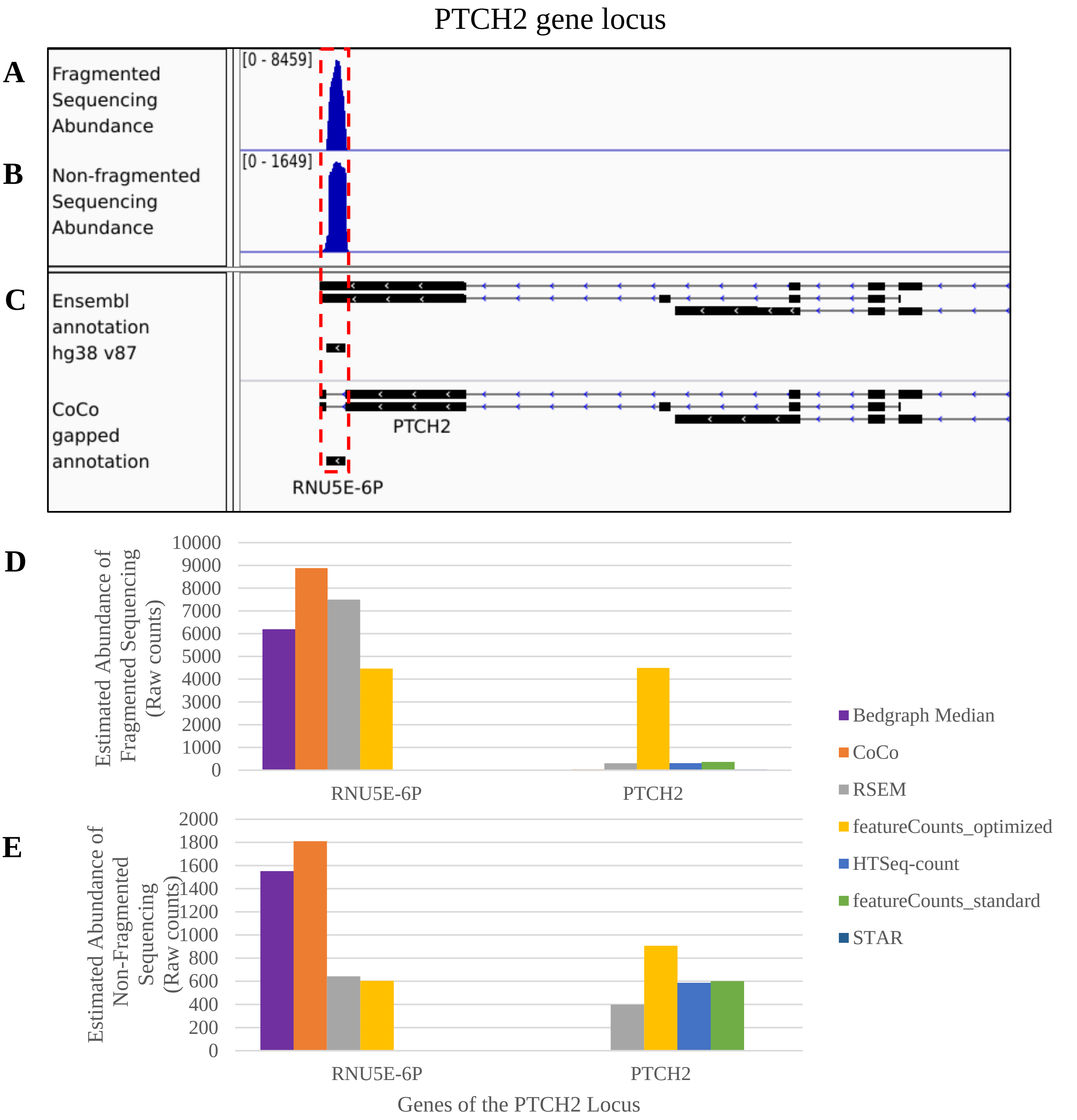

Supplementary Figure 4 : Example of a nested snRNA in TGIRT-seq fragmented and non-fragmented datasets. (A,B) Bedgraph screenshot of the PTCH2 gene locus which encodes the non-coding gene PTCH2 and the nested snRNA U5 pseudogene RNU5E-6P. The nested gene is highlighted in a dashed red box. The upper track (panel A) is from a fragmented sequencing library while the lower track (panel B) is from a non-fragmented sequencing library. Panel C shows the annotation tracks including the original Ensembl annotation and the CoCo gapped annotation highlighting nested genes. (D,E) Raw counts obtained using the different read assignment tools considered compared to the bedgraph median values calculated, for the fragmented (D) and the non-fragmented (E) datasets. The bedgraph median is only given for the small non-coding RNAs since it is not representative of the raw counts for longer genes.

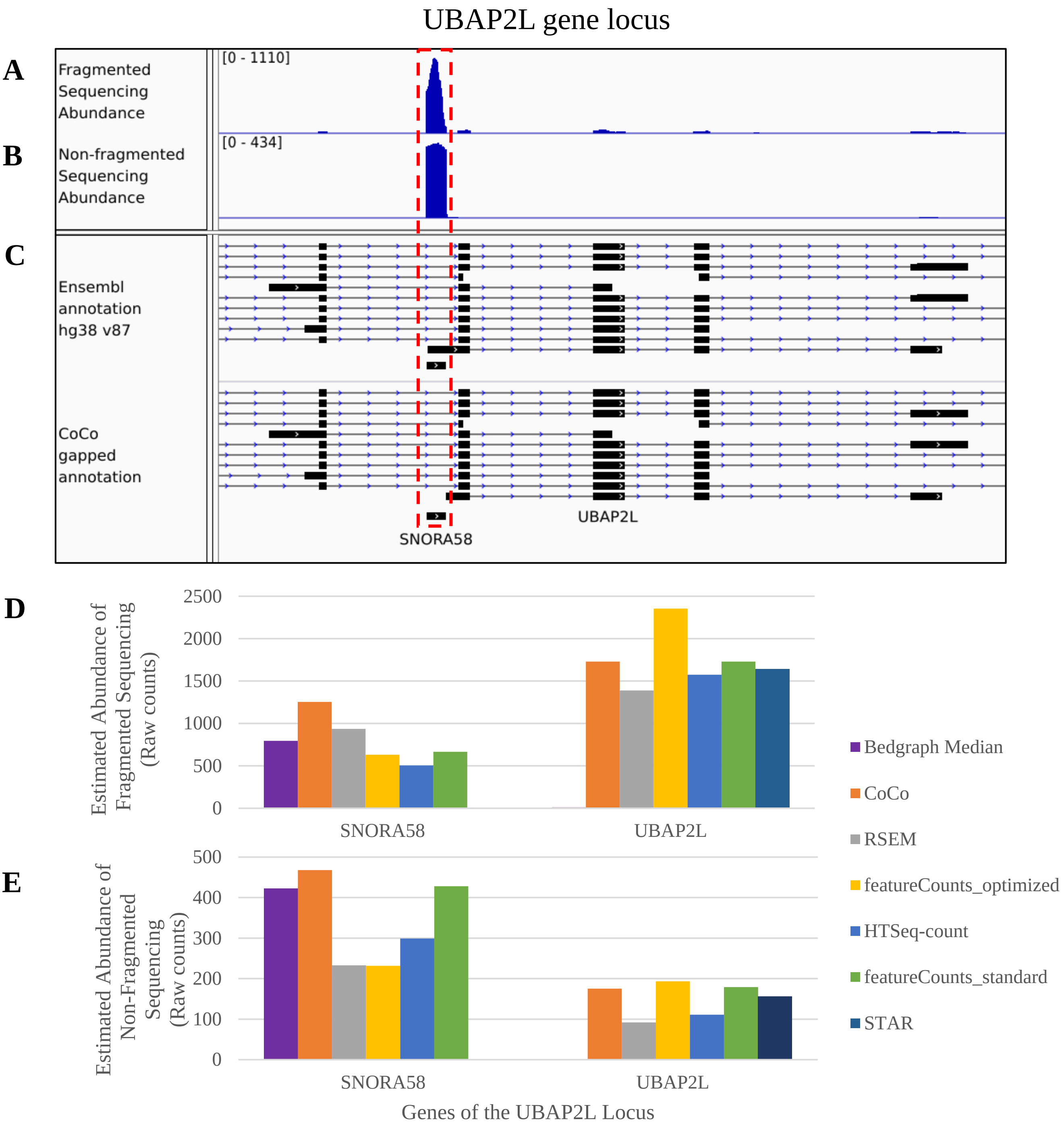

Supplementary Figure 5: Example of a nested H/ACA snoRNA in TGIRT-seq fragmented and non-fragmented datasets. (A,B) Bedgraph screenshot of the UBAP2L gene locus which encodes the coding gene UBAP2L and the nested box H/ACA snoRNA SNORA58. The nested gene is highlighted in a dashed red box. The upper track (panel A) is from a fragmented sequencing library while the lower track (panel B) is from a non-fragmented sequencing library. Panel C shows the annotation tracks including the original Ensembl annotation and the CoCo gapped annotation highlighting nested genes. (D,E) Raw counts obtained using the different read assignment tools considered compared to the bedgraph median values calculated, for the fragmented (D) and the non-fragmented (E) datasets. The bedgraph median is only given for the small non-coding RNAs since it is not representative of the raw counts for longer genes.

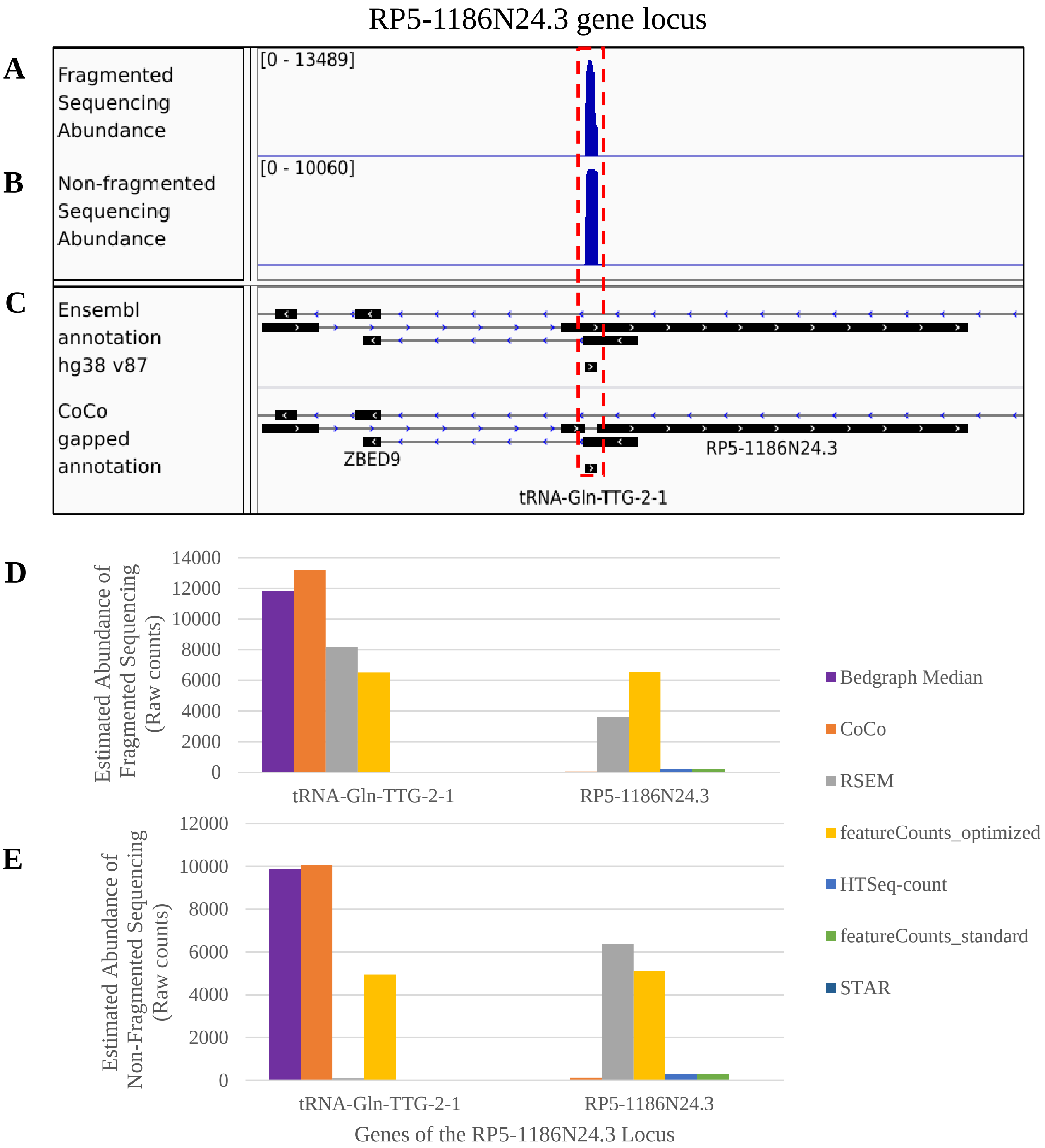

Supplementary Figure 6: Example of a nested tRNA in TGIRT-seq fragmented and non-fragmented datasets. (A,B) Bedgraph screenshot of the RP5-1186N24.3 gene locus which encodes the non-coding gene RP5-1186N24.3 and the nested tRNA tRNA-Gln-TTG-2-1. The nested gene is highlighted in a dashed red box. The upper track (panel A) is from a fragmented sequencing library while the lower track (panel B) is from a non-fragmented sequencing library. Panel C shows the annotation tracks including the original Ensembl annotation and the CoCo gapped annotation highlighting nested genes. (D,E) Raw counts obtained using the different read assignment tools considered compared to the bedgraph median values calculated, for the fragmented (D) and the non-fragmented (E) datasets. The bedgraph median is only given for the small non-coding RNAs since it is not representative of the raw counts for longer genes.

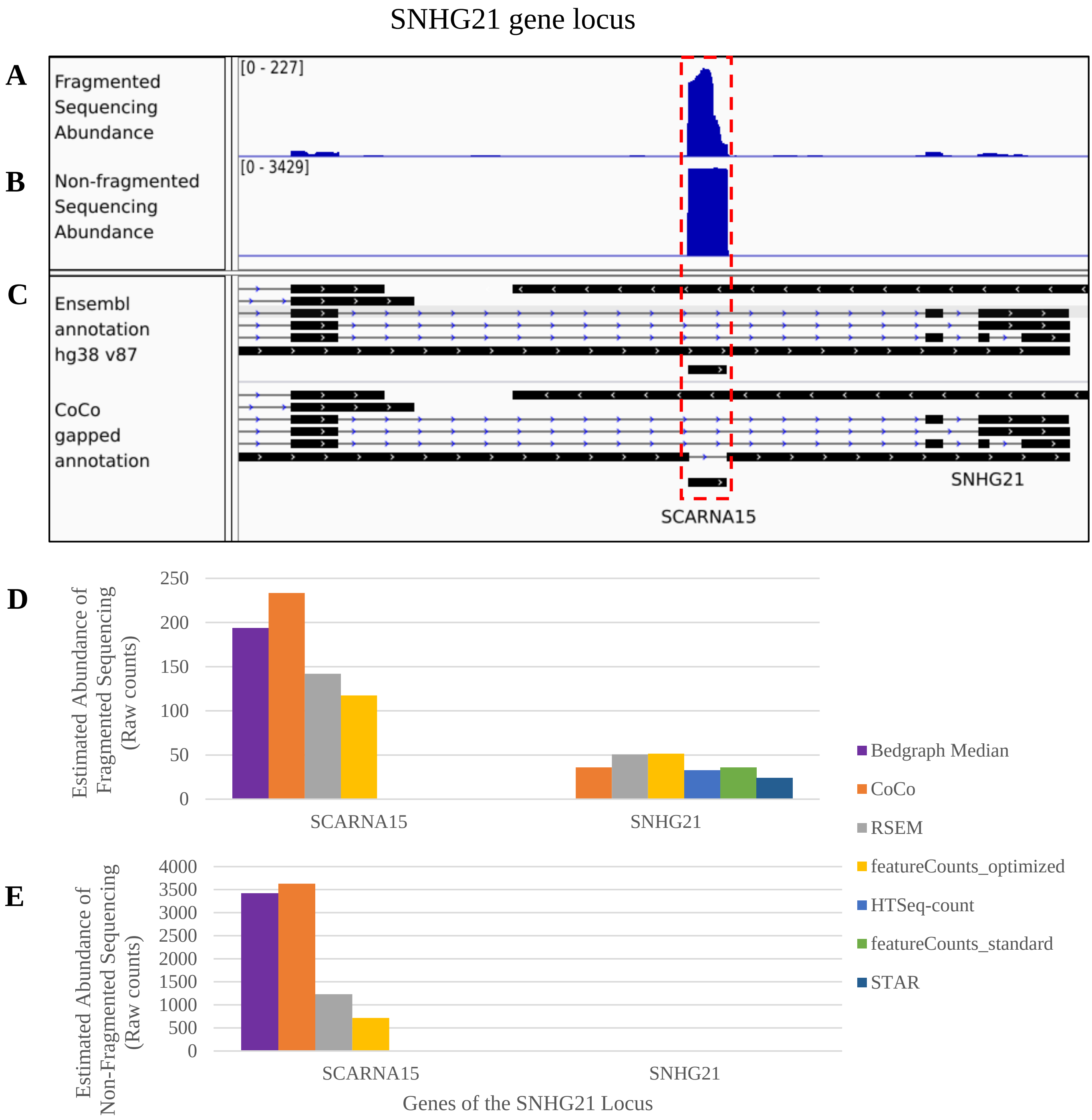

Supplementary Figure 7: Example of a nested scaRNA in TGIRT-seq fragmented and non-fragmented datasets. (A,B) Bedgraph screenshot of the SNHG21 gene locus which encodes the non-coding gene SNHG21 and the nested scaRNA SCARNA15. The nested gene is highlighted in a dashed red box. The upper track (panel A) is from a fragmented sequencing library while the lower track (panel B) is from a non-fragmented sequencing library. Panel C shows the annotation tracks including the original Ensembl annotation and the CoCo gapped annotation highlighting nested genes. (D,E) Raw counts obtained using the different read assignment tools considered compared to the bedgraph median values calculated, for the fragmented (D) and the non-fragmented (E) datasets. The bedgraph median is only given for the small non-coding RNAs since it is not representative of the raw counts for longer genes.

LINC00324 gene locus

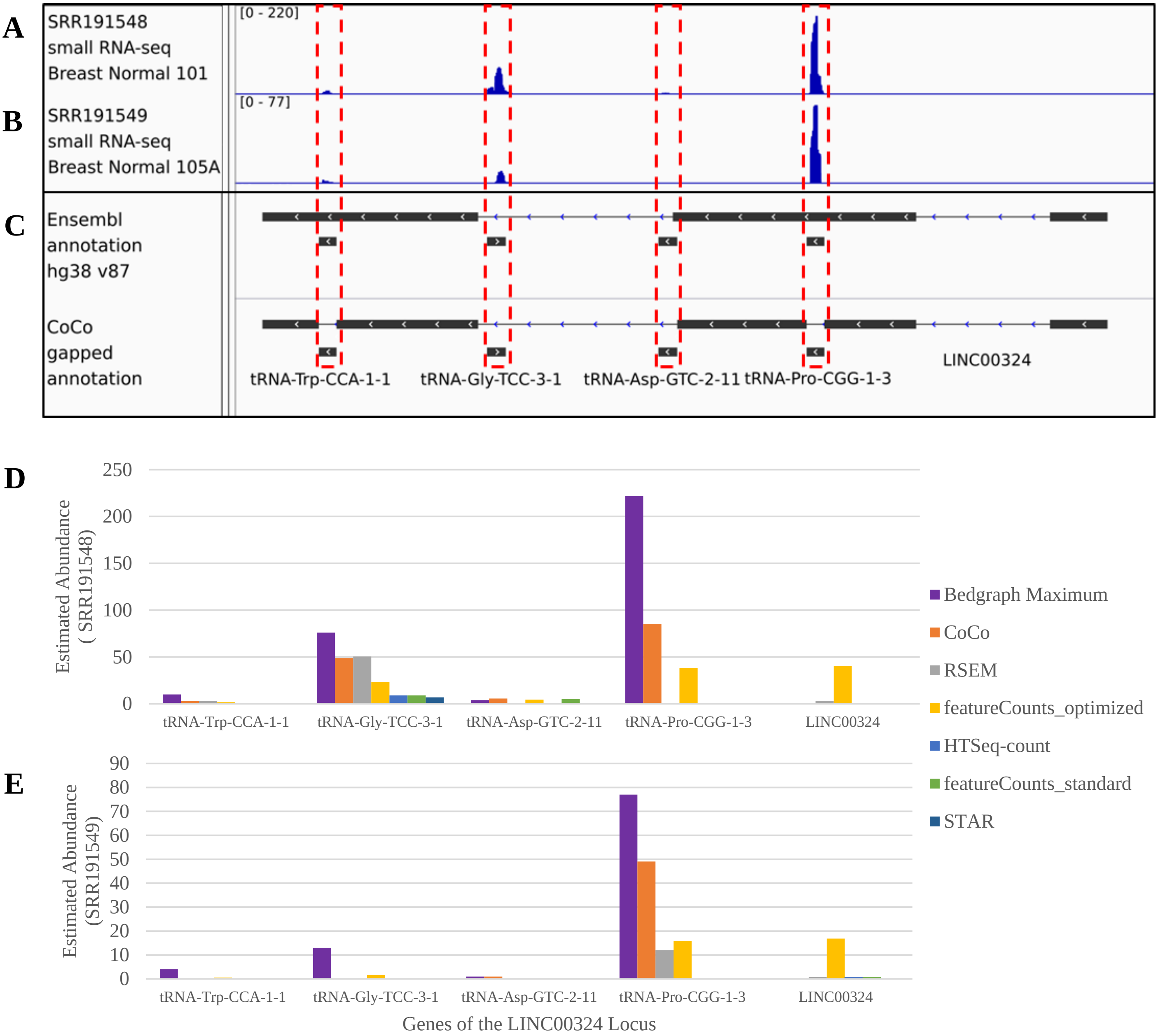

Supplementary Figure 8: **Example of nested tRNAs in small RNA-seq datasets.** (A,B) Bedgraph screenshot of LINC00324 gene locus which encodes the non-coding gene LINC00324 and the nested tRNA-Trp-CCA-1-1, tRNA-Gly-TCC-3-1, tRNA-Asp-GTC-2-11 and tRNA-Pro-CGG-1-3. The nested genes are highlighted in dashed red boxes. The upper tracks are barcoded small RNA-seq datasets from (Farazi et al, 2011) [a]: (A) SRR191548 and (B) SRR191549. Panel C shows the annotation tracks including the original Ensembl annotation and the CoCo gapped annotation highlighting nested genes. (D, E) Raw counts obtained using the different read assignment tools considered compared to the bedgraph maximum values calculated, for the SRR191548 (D) and the SRR191549 (E) datasets. The bedgraph maximum is only given for the small non-coding RNAs since it is not representative of the raw counts for longer genes.

[a] Farazi T.A., et al. (2011). MicroRNA Sequence and Expression Analysis in Breast Tumors by Deep Sequencing. *Cancer Res*, 71, 4443–4453.

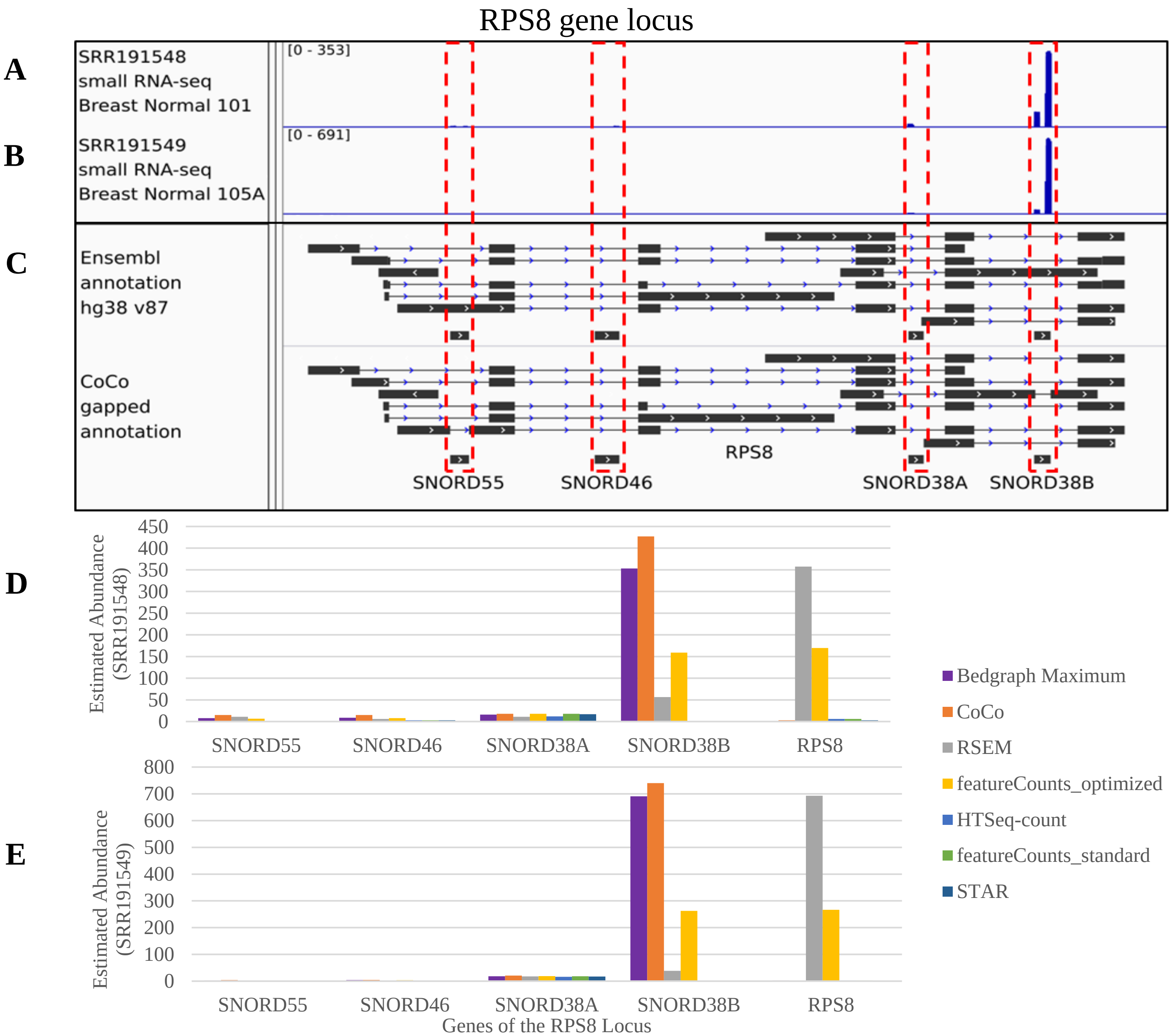

Supplementary Figure 9: **Example of nested box C/D snoRNAs in small RNA-seq datasets.** (A,B) Bedgraph screenshot of RPS8 gene locus which encodes the coding gene RPS8 and the nested box C/D snoRNAs SNORD55, SNORD46, SNORD38A and SNORD38B. The nested genes are highlighted in dashed red boxes. The upper tracks are barcoded small RNA-seq datasets from (Farazi et al, 2011)[a] : (A) SRR191548 and (B) SRR191549. Panel C shows the annotation tracks including the original Ensembl annotation and the CoCo gapped annotation highlighting nested genes. (D, E) Raw counts obtained using the different read assignment tools considered compared to the bedgraph maximum values calculated, for the SRR191548 (D) and the SRR191549 (E) datasets. The bedgraph maximum is only given for the small non-coding RNAs since it is not representative of the raw counts for longer genes. The estimated abundance is expected to be higher than the bedgraph maximum because short reads can align to different regions of the gene (for example, two peaks of accumulation are seen for SNORD38B). The estimated abundance should be the sum of the abundance of both expressed regions.

[a] Farazi T.A., et al. (2011). MicroRNA Sequence and Expression Analysis in Breast Tumors by Deep Sequencing. *Cancer Res*, 71, 4443–4453.

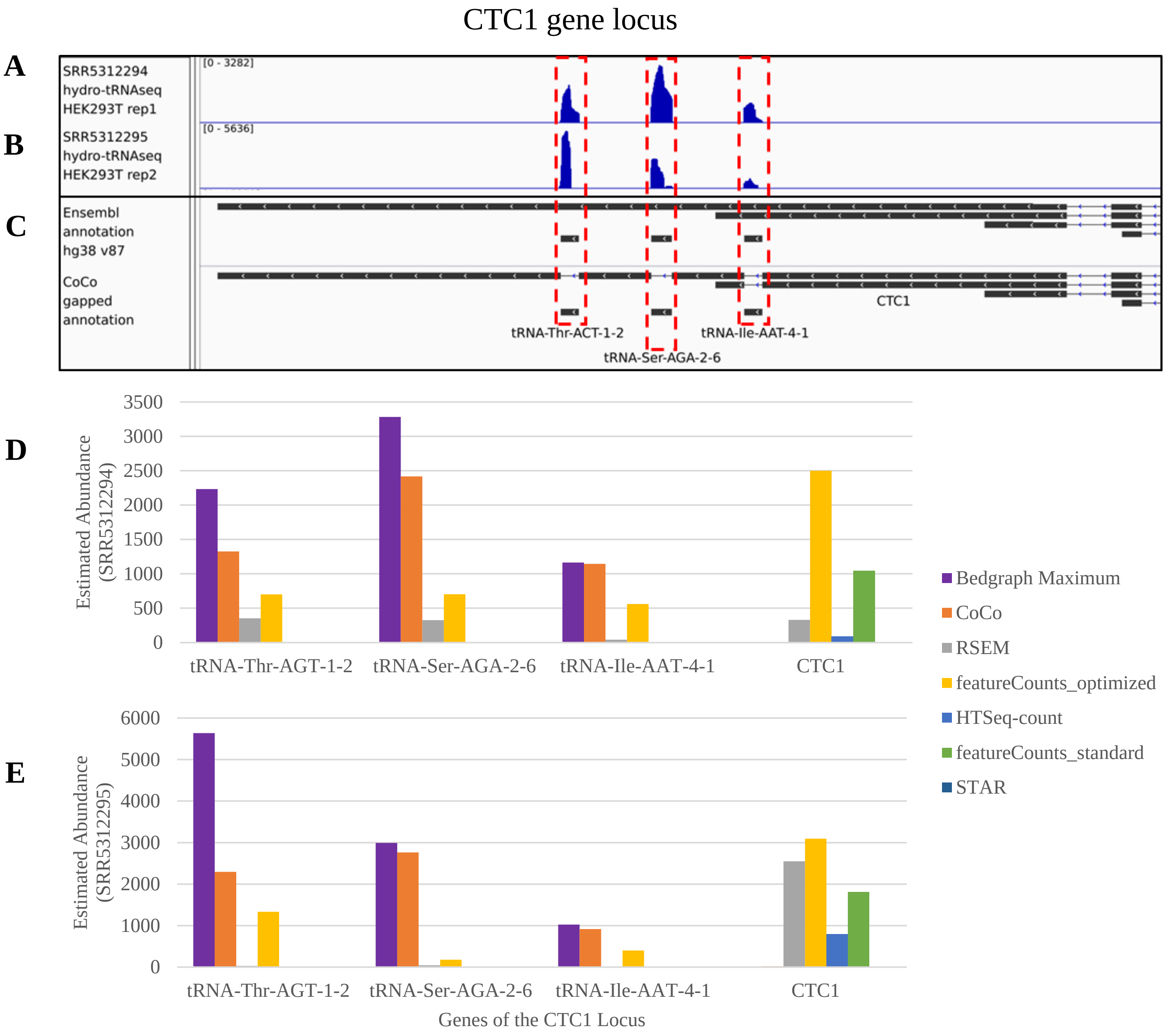

Supplementary Figure 10: **Example of nested tRNAs in hydro-tRNAseq datasets.** (A,B) Bedgraph screenshot of CTC1 gene locus which encodes the coding gene CTC1 and the nested tRNAs tRNA-Thr-AGT-1-2, tRNA-Ser-AGA-2-6 and tRNA-Ile-AAT-4-1. The nested genes are highlighted in dashed red boxes. The upper tracks are hydro-tRNAseq datasets from (Gogakos et al, 2017) [a] : (A) SRR5312294 and (B) SRR5312295. Panel C shows the annotation tracks including the original Ensembl annotation and the CoCo gapped annotation highlighting nested genes. (D, E) Raw counts obtained using the different read assignment tools considered compared to the bedgraph maximum values calculated, for the SRR5312294 (D) and the SRR5312295 (E) datasets. The bedgraph maximum is only given for the small non-coding RNAs since it is not representative of the raw counts for longer genes.

[a] Gogakos, T., Brown, M., Garzia, A., Meyer, C., Hafner, M., and Tuschl, T. (2017). Characterizing Expression and Processing of Precursor and Mature Human tRNAs by Hydro-tRNAseq and PAR-CLIP. *Cell Rep*, **20**, 1463–1475.

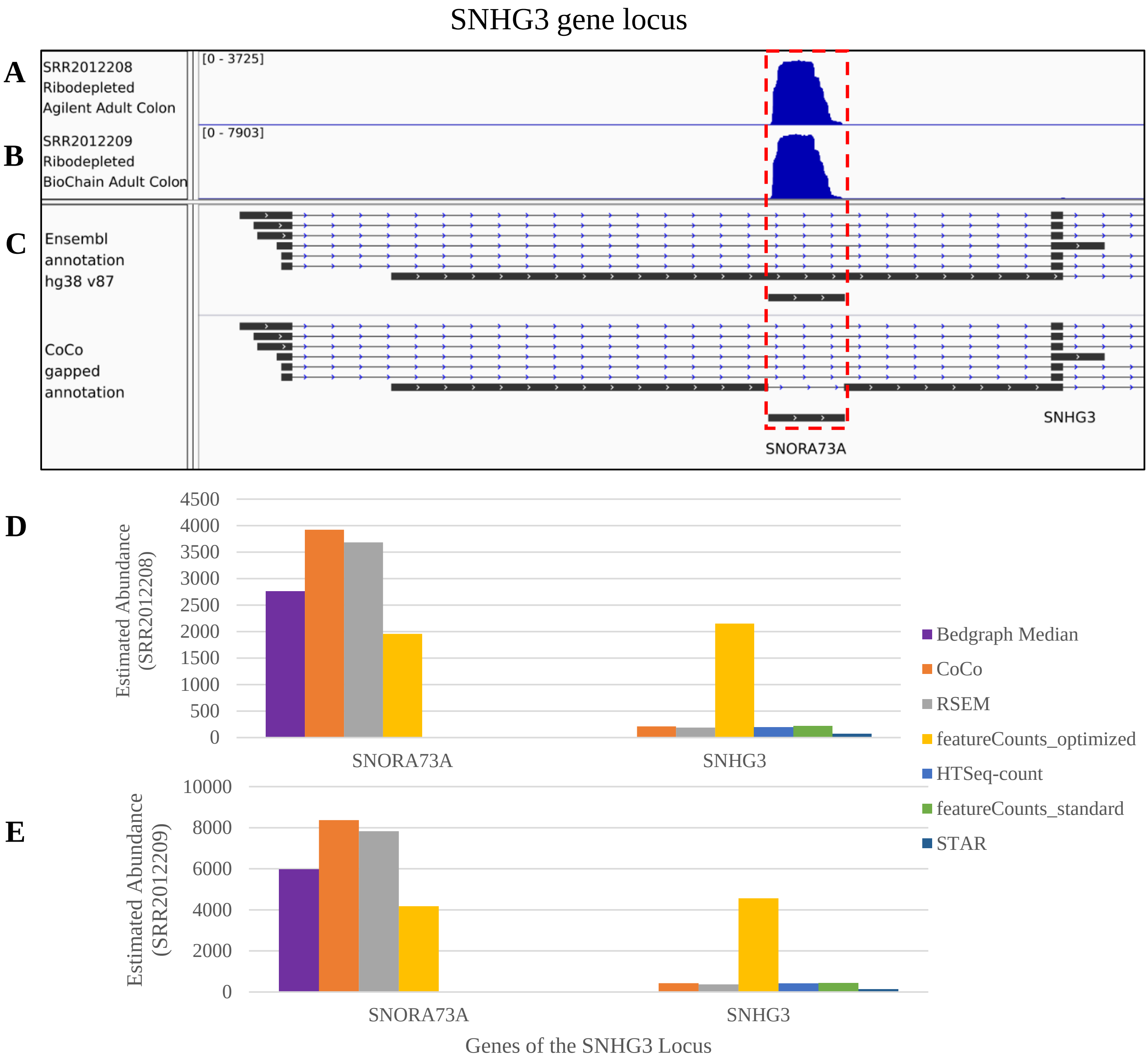

Supplementary Figure 11: **Example of nested H/ACA snoRNAs in standard viral RT ribodepleted RNA-seq datasets.** (A,B) Bedgraph screenshot of the SNHG3 gene locus which encodes the non-coding gene SNHG3 and the nested box H/ACA snoRNAs SNORA73A. The nested genes are highlighted in dashed red boxes. The upper tracks are standard ribodepleted datasets from (Choy et al, 2015)[a] : (A) SRR2012208 and (B) SRR2012209. Panel C shows the annotation tracks including the original Ensembl annotation and the CoCo gapped annotation highlighting nested genes. (D, E) Raw counts obtained using the different read assignment tools considered compared to the bedgraph median values calculated, for the SRR2012208 (D) and the SRR2012209 (E) datasets. The bedgraph median is only given for the small non-coding RNAs since it is not representative of the raw counts for longer genes.

[a] Choy, J.Y.H., Boon, P.L.S., Bertin, N., and Fullwood, M.J. (2015). A resource of ribosomal RNA-depleted RNA-Seq data from different normal adult and fetal human tissues. *Sci. Data* , **2**, 150063.

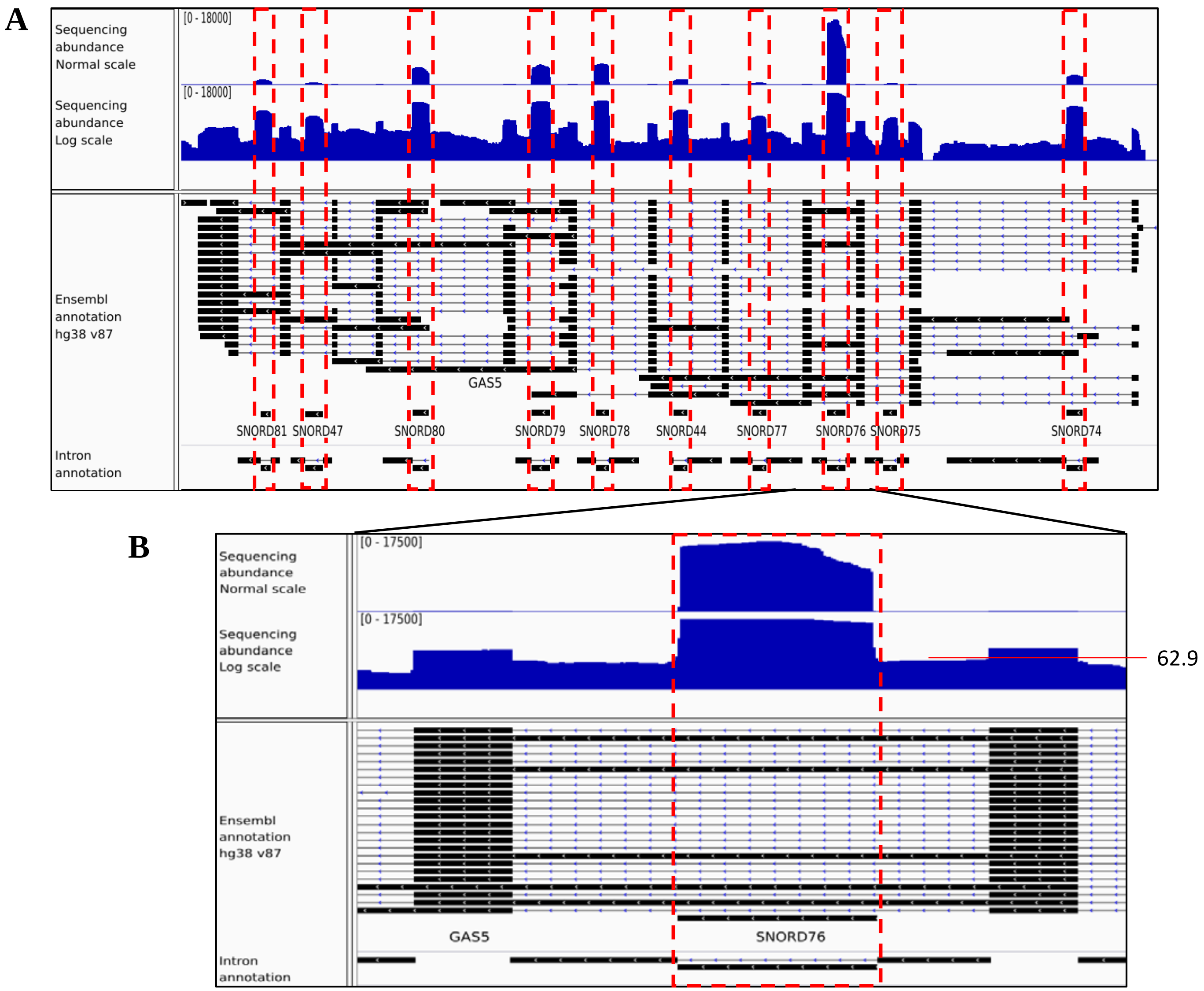

Supplementary Figure 12: Example of the background correction for nested genes from the GAS5 locus from a TGIRT-seq fragmented dataset (continued on next page).

C

Gene	Gene ID	Before Intron Correction	Number of Reassigned Reads	After Intron Correction
SNORD81	NR_003938	1344	-37.1	1306.9
SNORD47	NR_002746	690	-43.8	646.2
SNORD80	NR_003940	4670	-70.4	4599.6
SNORD79	NR_003939	5587	-52.2	5534.8
SNORD78	NR_003944	5674	-8.8	5665.3
SNORD44	NR_002750	1568	-16.5	1551.5
SNORD77	NR_003943	614	-21.2	592.8
SNORD76	NR_003942	17653	-62.9	17590.1
SNORD75	NR_003941	523	-16.6	506.4
SNORD74	NR_002579	2735	-9.1	2725.9
GAS5	ENSG00000234741	2049	313.2	2362.2

Supplementary Figure 12: Example of the background correction for nested genes from the GAS5 locus from a TGIRT-seq fragmented dataset (continued from previous page). (A) Screenshot of the GAS5 locus that encodes the lncRNA GAS5 and 10 box C/D snoRNAs. The top track shows the distribution of reads for the locus in normal scale while the second track shows the distribution of reads in log scale, to make apparent the background reads of the introns. The bottom tracks show the transcript, nested genes and intron annotations for this locus. The nested genes are highlighted in dashed red boxes. (B) Same as (A), but for a zoomed in region of the GAS5 locus, focusing on the nested gene SNORD76 and its flanking regions. While the SNORD76 nested gene obtains nearly a maximum of 17500 counts, the intronic regions flanking SNORD76 obtain on average 62.9 counts as shown in the log scale track. (C) Table indicating the read counts obtained for the nested and host genes before and after background correction. The background correction removes a fraction of the reads assigned to each nested gene and reassigns them to the host gene. Because the introns are detected at a very low level, the read counts obtained after the correction are not strongly different from the read counts calculated before the correction (compare the ‘Before Intron Correction’ column to the ‘After Intron Correction’ column).

A

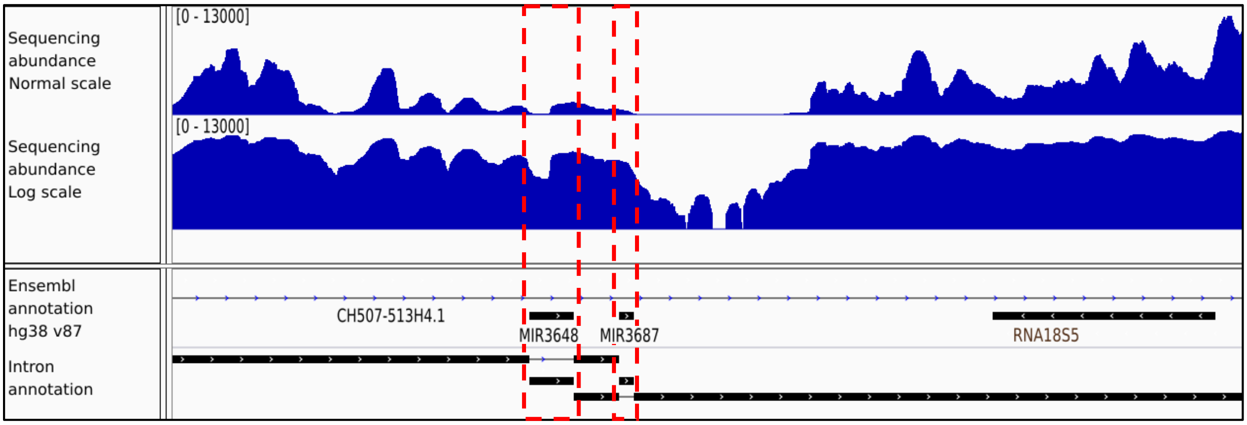

B

Gene	Gene ID	Before Intron Correction	Number of Reassigned Reads	After Intron Correction
MIR3648	ENSG00000275708	904	-904	0
MIR3687	ENSG00000277437	587	-587	0

Supplementary Figure 13: Example of the background correction for nested genes from the CH507-513H4.1 locus from a TGIRT-seq fragmented dataset. (A) Screenshot of the CH507-513H4.1 locus that encodes the lncRNA CH507-513H4.1 and 2 miRNAs, miR3648 and miR3687. The top track shows the distribution of reads for the locus in normal scale while the second track shows the distribution of reads in log scale, to make apparent the background reads of the introns. The bottom tracks show the transcript, nested genes and intron annotations for this locus. The nested genes are highlighted in dashed red boxes. (B) Table indicating the read counts obtained for the nested and host genes before and after background correction. Because the background levels are at least as high as the nested gene counts, the nested gene counts are corrected to 0 and these miRNAs are considered to be not detected in this experiment (compare the 'Before Intron Correction' column to the 'After Intron Correction' column).

A

```
SNORD103A   GAACTCATGAGCCCTTCTCAATTGAGGGGTTGGTTGGCCATTGTCTGGCAATGATGACCCACTTGCCCTCACTGAGAACAAAGTTC
SNORD103B   TGGGCTCTGAACTCTTCTCAATTGAGGGGTTGGTTGGCCATTGTCTGGCAATGATGACCCACTTGCCCTCACTGAGAACAAAGTTC
                   *** * *************************************************************************

SNORD103A   GGTAATGAGAATCTTTGTTAATGGACTCAAGTTCTGAGCCAGACAAGACACCCACCACCCCTGAGTCAAGCTAAACCAATCCAAA
SNORD103B   GGTAATGAGAATCTTTGTTAATGGACTCAAGTTCTGAGCCAGACAAGACACCCACCACCCCTGAGTCAAGCTAAACCAATCCAAA
            *************************************************************************************
```

B

Gene	Ratio of Uniquely Mapped Reads	Number of Multimapped Read Pairs	Distribution of Multimapped Read Pairs	Total Read Pair Counts
SNORD103A	50 (0.57)	2314	0.57*2314 = 1330	50+1330 = 1380
SNORD103B	37 (0.43)		0.43*2314 = 984	37+984 = 1021
Total	87 (1)	2314	2314	2401

C

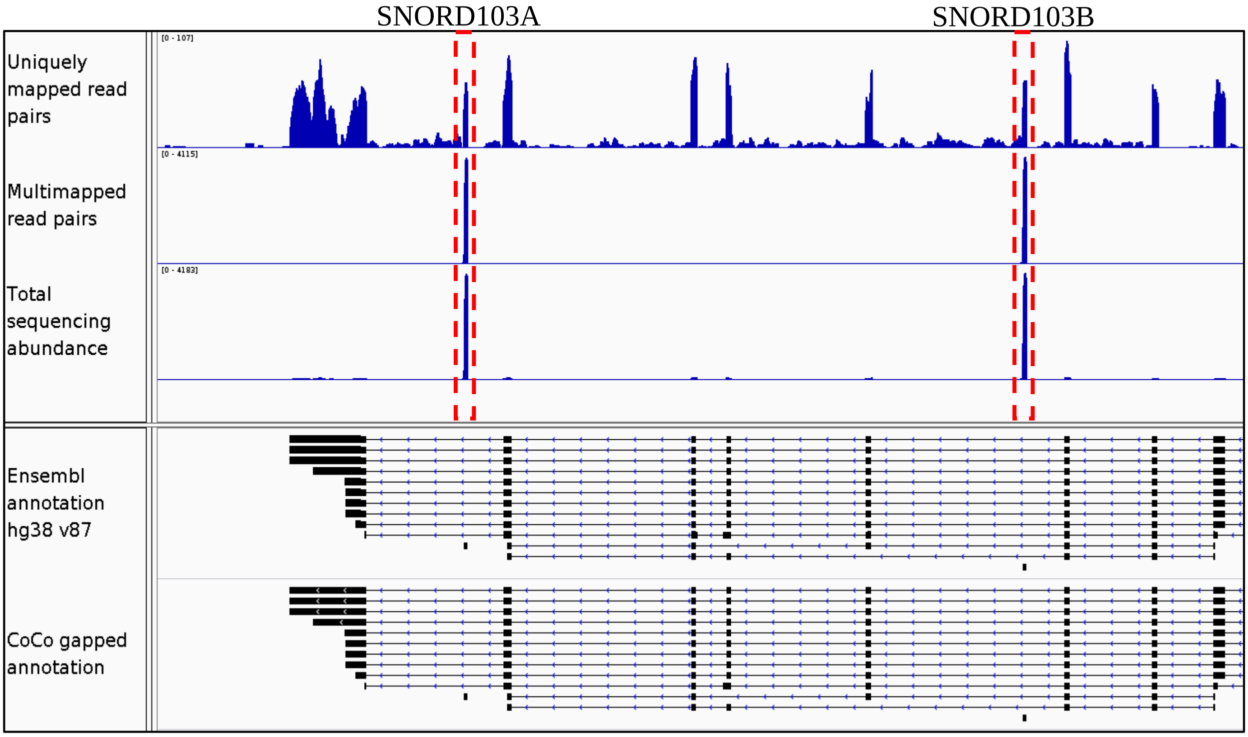

Supplementary Figure 14 : Example of CoCo based correction for multimapped read groups from a TGIRT-seq fragmented dataset. (**A**) Comparison of the genomic sequences of the box C/D snoRNAs SNORD103A and SNORD103B. SNORD103A and SNORD103B, located in two separate introns within their host gene PUM1, have identical mature sequence (shown in black) but non-identical genomic flanking sequences (shown in red, 40 nucleotides on either side). (**B**) Example of CoCo multimapped read distribution for fragmented datasets. CoCo considers the uniquely mapped counts for SNORD103A and SNORD103B and distributes the multimapped read pairs according to the uniquely mapped ratio. (**C**) Bedgraph showing the distribution of both unique and multimapped read pairs for SNORD103A and SNORD103B. The positions of the snoRNA reads are indicated on top (highlighted in dashed red boxes) and the Ensembl and CoCo gapped annotation are shown at bottom.

A

```
MIR101-1  --TGCCCTGGCTCAGTTATCACAGTGCTGATGCTGTCTATTCTAAAGGTACAGTACTGTGATAACTGAAGGATGGCA--
MIR101-2  ACTGTCCTTTTTCGGTTATCATGGTACCGATGCTGTATATCTGAAAGGTACAGTACTGTGATAACTGAAGAATGGTGGT
            ** ***   ** *******  ** * ******** ***   *************************** ****
```

B

C

D

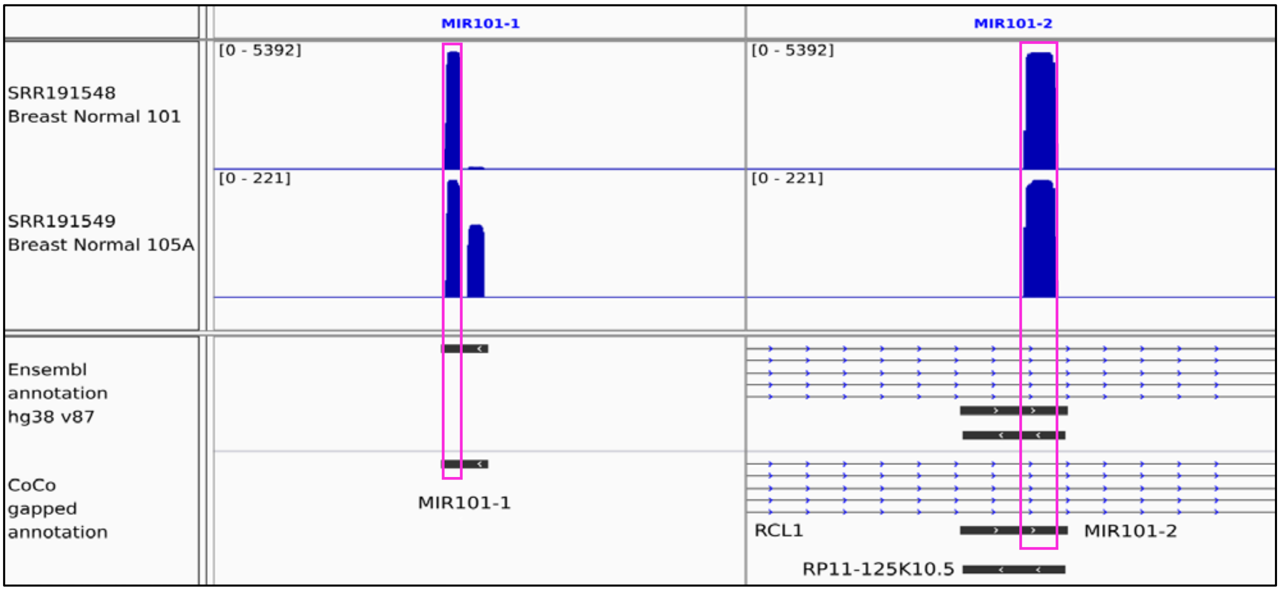

E

F

Dataset	gene_id	gene_name	CoCo	RSEM	featureCounts optimized	HTSeq-count	featureCounts standard	STAR
SRR191548	ENSG00000199065	MIR101-2	0	26.8	1347.22	0	0	11
	ENSG00000199135	MIR101-1	5495	4784.37	2811.69	103	160	99
	Total		5495	4811.17	4158.91	103	160	110
SRR191549	ENSG00000199065	MIR101-2	0	6.39	55.15	0	0	0
	ENSG00000199135	MIR101-1	361	350.61	245.99	134	137	134
	Total		361	357	301.14	134	137	134

Supplementary Figure 15: **Example of miRNA multimapped reads from small RNA-seq datasets.** (A) Comparison of the genomic sequences of the paralogues MIR101-1 and MIR101-2. The pink sequence corresponds to the expressed region shown in the bedgraph. This region is identical in the two paralogues. (B,C) Bedgraph screenshot of the MIR101-1 locus (left pane) and the RCL1 gene locus which encodes the coding gene RCL1 and the intronic miRNA MIR101-2 (right pane). The upper tracks are barcoded small RNA-seq datasets from (Farazi et al, 2011) [a]: (B) SRR191548 and (C) SRR191549. The identical region is highlighted in pink. All the reads in the pink region align to both genes and are represented in the bedgraph in both loci. Panel D shows the annotation tracks including the original Ensembl annotation and the CoCo gapped annotation highlighting nested genes. (E, F) Raw counts obtained using the different read assignment tools considered, for the SRR191548 (E) and the SRR191549 (F) datasets. The sum of both gene counts is given for the two datasets. The tools that don't consider the multimapped reads (HTSeq-count, featureCounts_standard and STAR) greatly underestimate these genes. CoCo and RSEM agree that most of the reads should be assigned to MIR101-1 because it has the most uniquely mapped reads as shown in the bedgraph by the expression outside of the pink region. The estimated abundance is expected to be higher than the bedgraph maximum because short reads can align to different regions of the gene (for example, two peaks corresponding the mature miRNA and the * miRNA are seen in panel C for MIR101-1). The estimated abundance should be the sum of the abundance of both expressed region.

[a] Farazi T.A., et al. (2011). MicroRNA Sequence and Expression Analysis in Breast Tumors by Deep Sequencing. *Cancer Res*, 71, 4443–4453.

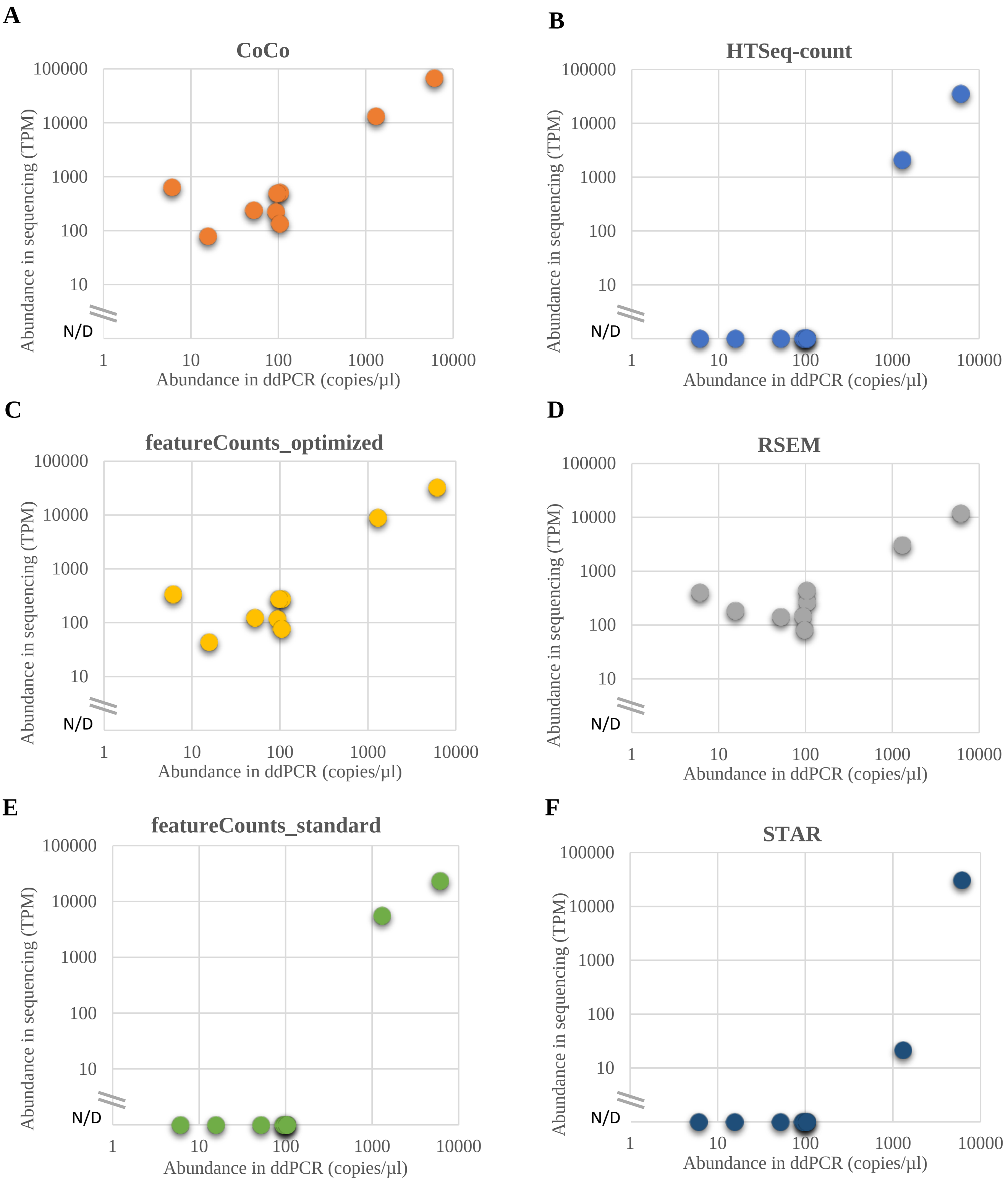

Supplementary Figure 16: Comparison between ddPCR and different read assignment tool estimates for chosen nested and multimapped genes. Shown are the estimated abundance of 9 nested or multimapped genes by fragmented TGIRT-seq as a function of ddPCR using the CoCo pipeline (A), HTSeq (B), featureCounts with optimized parameter values (C), RSEM (D), featureCounts with standard parameter values (E) and STAR (F). N/D in sequencing abundance stands for "Not Detected".

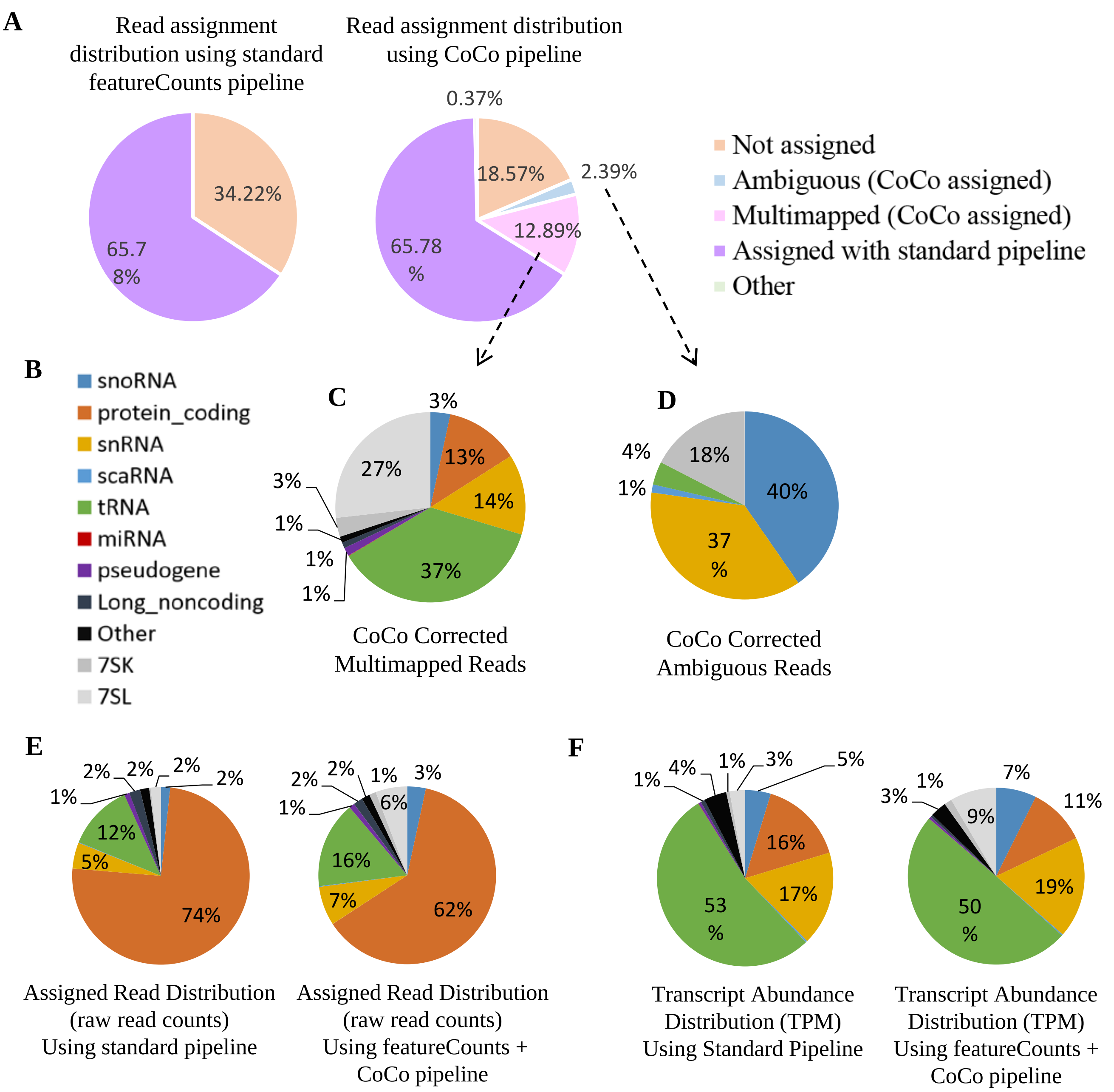

Supplementary Figure 17: Impact of the CoCo pipeline on the distribution of read assignments and the prediction of relative RNA abundance, for fragmented TGIRT-seq datasets. (**A**) Distribution of read assignment status as provided by the standard pipeline (left) or the CoCo pipeline (right). The color legend is shown to the right. Note that the ambiguous and multimapped reads are assigned only by CoCo. (**B**) Color legend for the pie charts in panels C-F representing the different biotypes of the genes to which read pairs were assigned. (**C**) Pie chart representing the distribution of the gene biotype to which were assigned multimapped reads by CoCo. (**D**) Pie chart representing the distribution of the gene biotype to which were assigned ambiguous reads by CoCo. (**E**) Distribution of the assigned raw read counts using the standard (left) or CoCo (right) pipelines by biotype. (**F**) Distribution of the normalized transcript estimates (TPM) assigned by the standard (left) or CoCo (right) pipelines by biotype.

A **B**

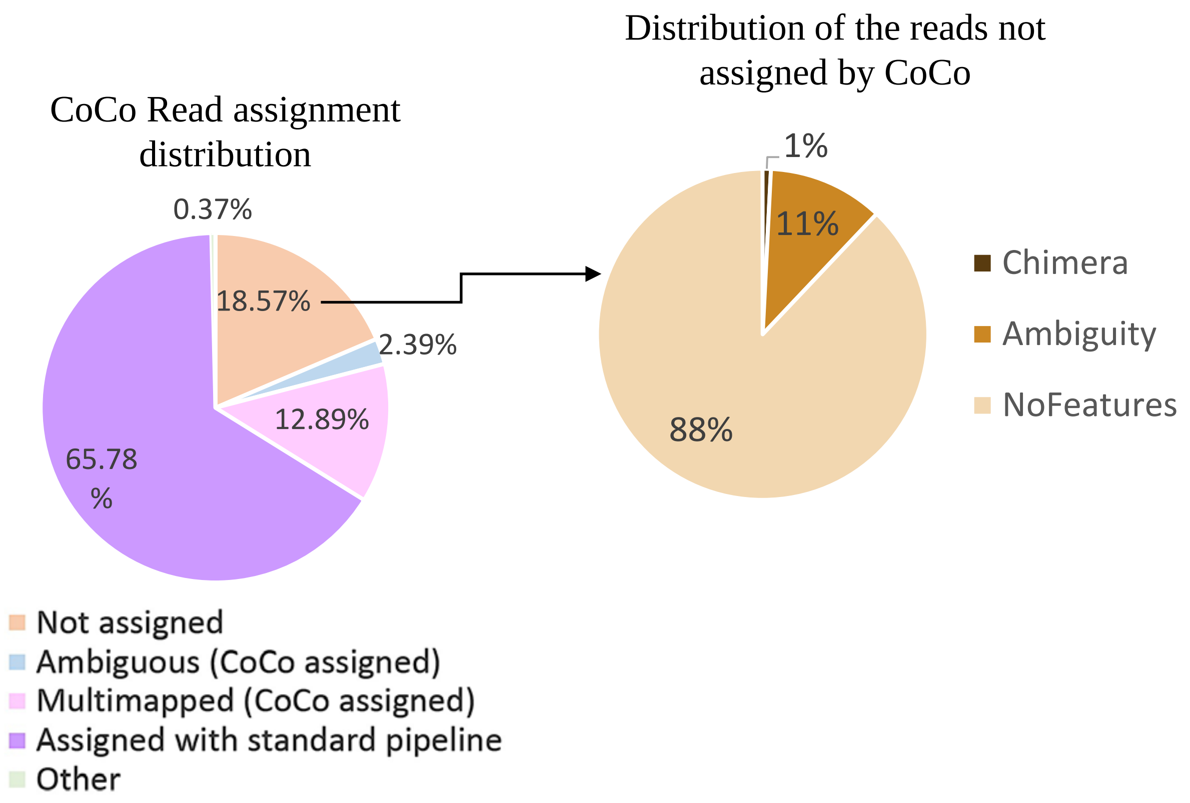

Supplementary Figure 18: Distribution of read pairs that are not assigned by CoCo, for fragmented TGIRT-seq datasets. (A) Pie chart illustrating the distribution of the CoCo assigned reads. Note that 18.6% of the reads are not assigned by CoCo. (B) A pie chart showing the type of reads not assigned by CoCo. The majority of the unassigned reads (88%) align to genomic regions in which no feature is annotated. The rest of the unassigned reads are either ambiguous (aligning to multiple features) or chimeric reads.

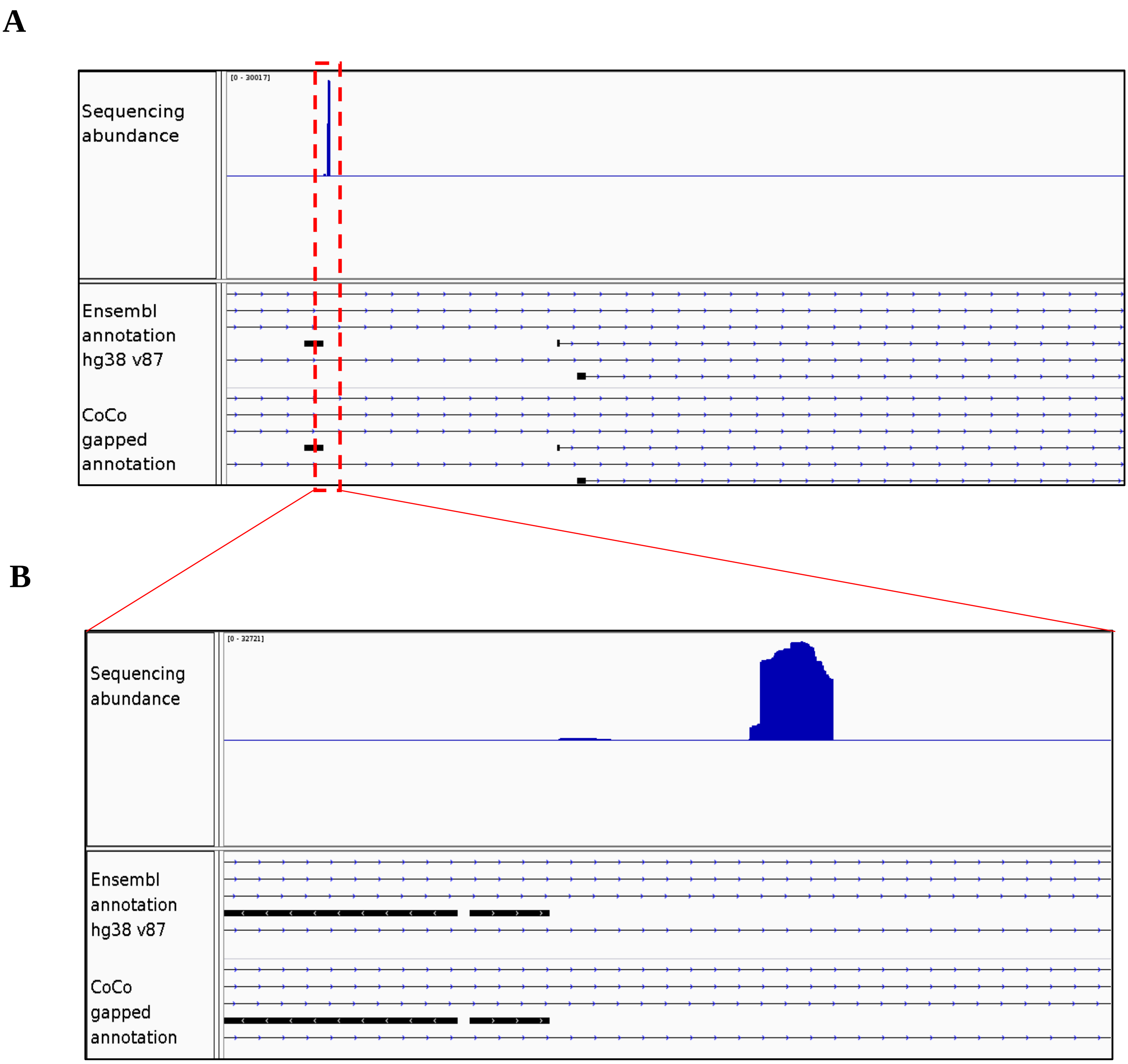

Supplementary Figure 19: Example of reads generated from unannoted sequence in fragmented TGIRT-seq datasets. (**A**) Shown is the bedgraph illustrating the reads accumulating in a featureless region (i.e. region with no annotated feature) within the intron of the AKAP6 gene. (**B**) Magnification of AKAP6 intronic region generating the majority of the featureless sequence reads. Note the lack of reads in the adjacent exonic sequence.

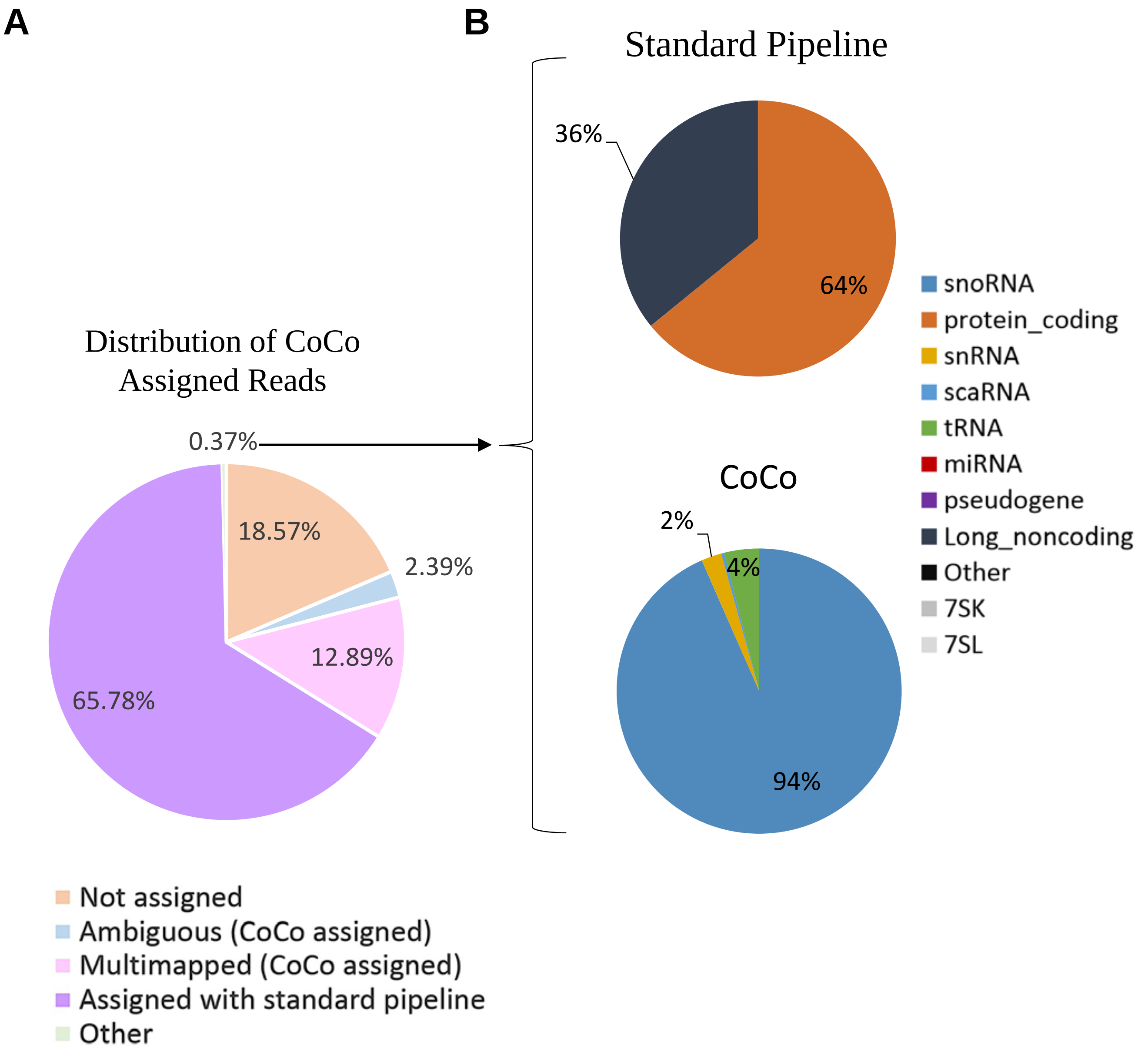

Supplementary Figure 20: Distribution of the read pairs that are differentially assigned by CoCo, for fragmented TGIRT-seq datasets. (**A**) Coco based read distribution. The reads detected by Coco are shown in the form of a pie chart. (**B**) Distribution of the reads that are differentially assigned by CoCo and standard pipelines (featureCounts alone). The reads that are differentially assigned by the different pipelines, which represent the majority of the read pairs annotated as 'Other' in panel A, are shown in the form of pie charts for either the standard pipeline (upper panel) or to the CoCo pipeline (bottom panel). Note that most reads that are assigned to genes of biotype protein coding or lncRNA by the standard pipeline are assigned to snoRNA, tRNAs and snRNAs by CoCo.

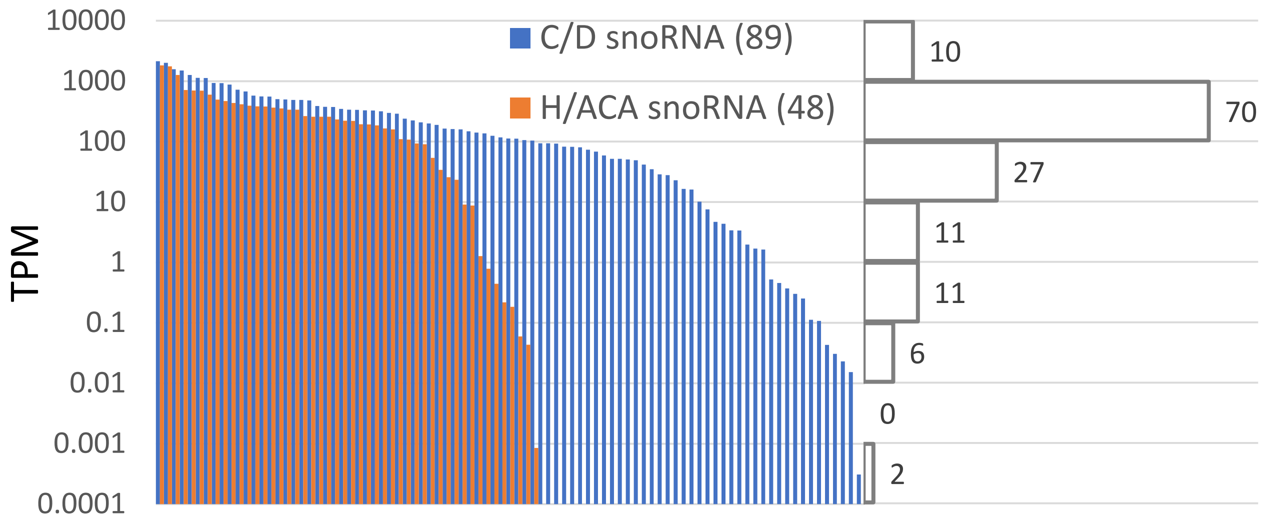

Supplementary Figure 21: Distribution of the corrected abundance of the 137 snoRNAs detected using the CoCo pipeline but not using a standard read assignment pipeline, for fragmented TGIRT-seq datasets. The abundance for each of the 89 C/D and 48 H/ACA box snoRNA only detected by CoCo is shown as a bar chart on the left and their cumulative counts for TPM ranges is shown with bins in the horizontal histogram on the right.
